# Aspartate–Glutamate Carrier 1 (*SLC25A12*) Deficiency: Malate–Aspartate Shuttle Failure, Neurodevelopmental Epileptic Encephalopathy, and Ketone-Based Metabolic Therapy

**DOI:** 10.3390/ijms27104455

**Published:** 2026-05-15

**Authors:** Manuela Murano, Giorgia Natalia Iaconisi, Magnus Monné, Amer Ahmed, Giuseppe Fiermonte, Loredana Capobianco, Vincenza Dolce

**Affiliations:** 1 Department of Pharmacy, Health, and Nutritional Sciences, University of Calabria, Arcavacata di Rende, 87036 Cosenza, Italy; manuela.murano@unical.it; 2 Department of Biosciences, Biotechnologies and Environment, University of Bari, 70125 Bari, Italy; giorgia.iaconisi@uniba.it (G.N.I.); aa.biotechiub@gmail.com (A.A.); giuseppe.fiermonte@uniba.it (G.F.); 3 Department of Health Sciences, University of Basilicata, 85100 Potenza, Italy; magnus.monne@unibas.it; 4 Department of Biological and Environmental Sciences and Technologies, University of Salento, 73100 Lecce, Italy

**Keywords:** *SLC25A12*, aspartate/glutamate carrier 1 (AGC1), ketogenic diet, epilepsy, myelination, N-acetylaspartate (NAA)

## Abstract

Aspartate–glutamate carrier 1 (AGC1) deficiency is a rare neurometabolic disorder caused by biallelic pathogenic variants in *SLC25A12*. Clinically, it is characterized by early-onset developmental and epileptic encephalopathy, often associated with hypomyelination and reduced brain N-acetylaspartate. AGC1 loss reduces malate–aspartate shuttle flux, limiting cytosolic NAD^+^ regeneration and impairing neuronal redox coupling, ATP supply, and aspartate-dependent biosynthesis during brain development. We integrate human genetics with mechanistic evidence from mammalian, *Drosophila melanogaster*, and *Saccharomyces cerevisiae* models to describe conserved transport principles and species-specific regulation underlying selective central nervous system vulnerability. We review the management of AGC1 deficiency, focusing on ketogenic therapy. Published reports show reproducible seizure reduction and, in some patients, improved myelination and N-acetylaspartate. However, these responses are heterogeneous and appear to depend on the timing, duration, and stability of ketosis. Preclinical evidence suggests that β-hydroxybutyrate may contribute to metabolic support in AGC1 deficiency. Prospective studies should test disease modification using standardized endpoints plus MRI/^1^H-MRS and ketosis measures.

## 1. Introduction

Aspartate–glutamate carrier 1 (AGC1) deficiency is a rare neurometabolic disorder caused by biallelic pathogenic variants in *SLC25A12*, clinically characterized by early-onset developmental and epileptic encephalopathy, prominent neurodevelopmental impairment, and frequent global cerebral hypomyelination, often accompanied by reduced brain N-acetylaspartate (NAA) on proton magnetic resonance spectroscopy (^1^H-MRS) [1]. At the molecular level, AGC1 dysfunction compromises mitochondrial aspartate–glutamate exchange and disrupts the malate–aspartate shuttle (MAS), limiting cytosolic NADH reoxidation and thereby constraining neuronal redox balance and energy metabolism [2,3,4]. Because the developing brain relies heavily on tightly regulated redox coupling and aspartate-dependent biosynthetic fluxes, impaired AGC1 activity provides a coherent mechanistic framework linking mitochondrial transport failure to seizures, developmental delay, and white matter abnormalities.

AGC1 belongs to the mitochondrial carrier (MC) family, also known as the solute carrier family 25 (SLC25), the largest group of inner mitochondrial membrane transporters conserved across eukaryotes [5,6,7,8]. SLC25 carriers mediate the exchange of a broad range of metabolites, including carboxylates, amino acids, nucleotides, and cofactors, supporting oxidative phosphorylation, intermediary metabolism, and biosynthesis [9,10]. While most carriers operate as antiporters, selected members show distinct transport modes and additional regulatory domains tailored to tissue-specific metabolic demands [9,10,11,12].

**Figure 1:**
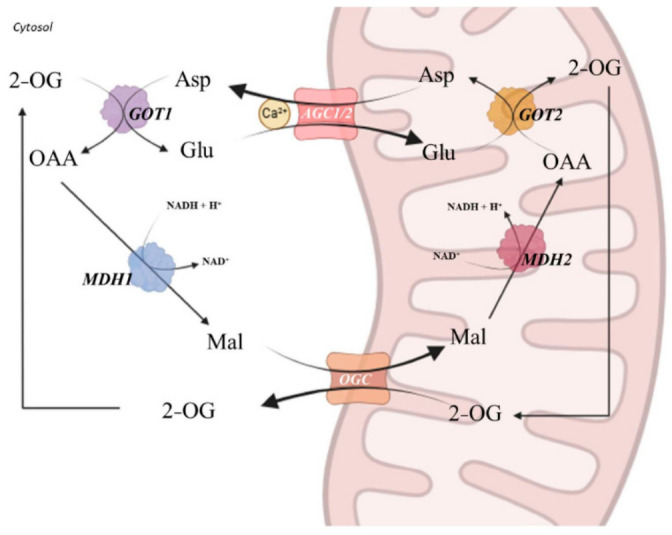
The malate–aspartate shuttle mediated by AGC1/2. The malate–aspartate shuttle transfers cytosolic reducing equivalents into mitochondria through a coordinated network of enzymatic reactions and mitochondrial carriers. In the cytosol, oxaloacetate (OAA) is reduced to malate (Mal) by malate dehydrogenase 1 (MDH1), consuming NADH. Mal is exchanged for 2-oxoglutarate (2-OG) across the inner mitochondrial membrane via 2-oxoglutarate carrier (OGC). In the mitochondrial matrix, malate dehydrogenase 2 (MDH2) oxidizes Mal to OAA, regenerating NADH. OAA is transaminated to aspartate (Asp) by mitochondrial aspartate aminotransferase (GOT2), using glutamate (Glu) as an amino donor. Asp is exported to the cytosol in exchange for Glu by the Ca^2+^-regulated aspartate–glutamate carriers AGC1 and AGC2, where cytosolic GOT1 completes the cycle. This shuttle is essential for maintaining cytosolic NAD^+^/NADH redox balance and cellular energy metabolism. Abbreviations: AGC1/2, aspartate–glutamate carriers 1 and 2; OGC, 2-oxoglutarate carrier; GOT1/2, cytosolic/mitochondrial aspartate aminotransferase; MDH1/2, cytosolic/mitochondrial malate dehydrogenase; OAA, oxaloacetate; Mal, malate; Asp, aspartate; Glu, glutamate; 2-OG, 2-oxoglutarate; NAD^+^/NADH, nicotinamide adenine dinucleotide (oxidized/reduced forms); Ca^2+^, calcium ion. Created in BioRender. https://BioRender.com/51g517h (accessed on 26 April 2026).

**Figure 2:**
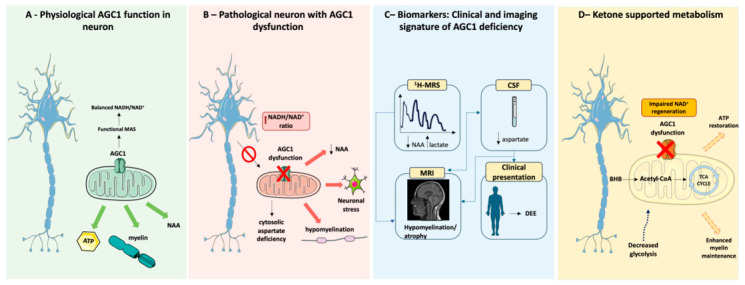
AGC1 (*SLC25A12*) deficiency: from impaired mitochondrial transport to brain phenotype. (**A**) Normal AGC1 function facilitates aspartate export, supporting N-acetylaspartate (NAA) synthesis, myelin maintenance, and ATP production. (**B**) AGC1 dysfunction impairs mitochondrial aspartate export, reducing cytosolic aspartate availability and disrupting malate–aspartate shuttle flux, with consequent cytosolic redox imbalance (increased NADH/NAD^+^), decreased N-acetylaspartate (NAA), neuronal metabolic stress, and secondary hypomyelination. (**C**) Clinical and imaging signatures include reduced NAA and elevated lactate levels on 1H-MRS, low aspartate in cerebrospinal fluid, hypomyelination and atrophy on MRI, and a clinical phenotype dominated by developmental and epileptic encephalopathy (DEE), often accompanied by spasticity and movement disorders. (**D**) Under ketogenic conditions, circulating ketone bodies, primarily β-hydroxybutyrate (BHB), are transported into the brain and metabolized by neurons to generate acetyl-CoA, which directly fuels mitochondrial oxidative metabolism and ATP production. By providing acetyl-CoA independently of glycolytic pyruvate metabolism, ketosis may reduce reliance on glycolysis and partially bypass the malate–aspartate shuttle (MAS), which is compromised in AGC1 deficiency. This metabolic adaptation may improve neuronal bioenergetic efficiency, contribute to redox homeostasis, and support myelin maintenance, thereby increasing metabolic flexibility in the AGC1-deficient brain. Adapted from Servier Medical Art “https://smart.servier.com (accessed on 27 April 2026)”, licensed under CC BY 4.0 “https://creativecommons.org/licenses/by/4.0/ accessed on 12 February 2026)”.

The aspartate–glutamate carriers (AGCs) are key MAS components and are encoded by *SLC25A12* (AGC1/aralar1) and *SLC25A13* (AGC2/citrin) [13]. AGCs catalyze an electrogenic exchange of cytosolic glutamate (and a proton) for mitochondrial aspartate, enabling regeneration of cytosolic NAD^+^ and sustained glycolytic flux [2,3,14]. The MAS function emerges from the coordinated activity of AGCs with additional transporters and enzymes, including the 2-oxoglutarate carrier (*OGC*), glutamic-oxaloacetic transaminases (GOT1/2), and malate dehydrogenases (MDH1/2) [5,9]. Notably, AGC1 and AGC2 are part of a calcium-regulated subfamily of mitochondrial carriers with N-terminal EF-hand motifs that couple cytosolic Ca^2+^ signals to carrier activity and metabolic flux [3,14,15] (Figure 1 and Figure 2A).

In reconstituted systems, AGC1 and AGC2 display essentially the same substrate spectrum and very similar transport affinities, with reported K_m_ values of about 50–56 µM for aspartate and 205–228 µM for glutamate [3]. These findings argue against major isoform-specific differences in transported metabolites and instead support the view that the two carriers perform a conserved core exchange reaction.

The main distinction between AGC1 and AGC2 therefore lies in tissue distribution and regulatory tuning rather than substrate specificity. AGC1/aralar is enriched in neurons and muscle, whereas AGC2/citrin is most abundant in the liver and other non-excitable tissues; the heart expresses both isoforms. The two carriers also differ in Ca^2+^ dependence, with citrin activating the malate-aspartate shuttle at lower Ca^2+^ concentrations than aralar (S0.5 about 100–150 nM for citrin versus about 280–350 nM for aralar) [13]. This partitioning likely allows mammals to adapt the same transport activity to different metabolic contexts. In the brain, aralar primarily supports neuronal MAS activity and is a key determinant of N-acetylaspartate (NAA) synthesis because the export of mitochondrial aspartate to the cytosol provides an essential substrate for NAA production [16]. This is particularly relevant to white matter pathology, since NAA is a major neuron-derived metabolite that supports myelination: its acetate moiety contributes to myelin lipid synthesis, and its metabolism in oligodendrocytes also supports energetic homeostasis during developmental myelination [17,18,19]. Accordingly, hypomyelination in AGC1 deficiency is likely to reflect not only impaired mitochondrial redox coupling and energy metabolism but also reduced AGC1-dependent NAA availability and diminished metabolic support for myelin formation [3,14,16,20]. By contrast, in the liver, citrin links MAS function to urea-cycle activity, gluconeogenesis, and hepatic redox balance [21,22,23,24]. Consistent with this specialization, *SLC25A12* deficiency causes a predominantly neurological phenotype with epilepsy, hypotonia, psychomotor arrest, and hypomyelination, whereas *SLC25A13* deficiency causes a predominantly hepatic/metabolic disorder that includes neonatal intrahepatic cholestasis and adult-onset type II citrullinemia [1,21,22,23,24]. Importantly, liver-specific expression of aralar can partially rescue redox/MAS defects in citrin-deficient hepatocytes, indicating substantial overlap in core transport function despite marked tissue specialization [25]. The main distinguishing features of the two mammalian aspartate-glutamate carriers, AGC1/aralar and AGC2/citrin, are summarized in Table 1.

Beyond the established Mendelian phenotype, common *SLC25A12* variants have also been investigated as susceptibility factors for autism spectrum disorder, although the overall evidence remains conflicting and is discussed separately [28,29].

In this review, we adopt a patient-centered perspective on AGC1 deficiency, integrating genetic and mechanistic insights with the reported clinical spectrum, neuroradiological/metabolic hallmarks, and therapeutic strategies, with particular emphasis on the ketogenic diet as a rational metabolic intervention.

## 2. Comparative AGC1 Function in Mammalian, Drosophila, and Yeast Models

Comparative studies of AGC1 orthologues clarify which aspects of transport and regulation are conserved and which have been adapted to organism-specific metabolic demands, providing a mechanistic framework relevant to the brain-selective vulnerability observed in AGC1 deficiency.

In mammals, AGC1 (Aralar1) is encoded by *SLC25A12* (2q24.3) and shows a highly tissue-selective pattern, with the strongest expression in energetically demanding tissues such as brain, heart, and skeletal muscle [1,14]. This enrichment in excitable tissues is consistent with the need for an efficient malate–aspartate shuttle (MAS) to maintain redox balance between cytosolic glycolysis and mitochondrial ATP production and, in neurons, to sustain myelin-related biosynthetic demands [24]. AGC1 operates as a Ca^2+^-regulated aspartate–glutamate antiporter, acting as a metabolic sensor via EF-hand Ca^2+^-binding motifs in its N-terminal domain [3,30]. By translating cytosolic Ca^2+^ signals into changes in carrier activity, AGC1 increases MAS flux and thereby supports mitochondrial NADH re-oxidation and ATP synthesis during periods of elevated energetic demand [3,30]. Transport properties of mitochondrial carriers are commonly determined using recombinant proteins expressed and reconstituted in liposomes [31,32]. Using this type of approach for AGC1, transport shows high affinity for aspartate (K_m_ ~51–52 µM) and glutamate (K_m_ ~220–229 µM), high transport capacity (V_max_ ~43–79 µmol/min/g for Asp/Asp and ~49–96 µmol/min/g for Glu/Asp), strict stereospecificity for L-isomers, and recognition of cysteinesulfinate [3]; these features are consistent with data obtained in isolated mouse liver mitochondria [33]. Functionally, Ca^2+^ binding to the cytosolic N-terminal EF-hands strongly potentiates transport, enhancing the aspartate–glutamate exchange and improving MAS efficiency without requiring Ca^2+^ uptake into the mitochondrial matrix [3,30]. In permeabilized HEK-293T cells, Ca^2+^ stimulation increases AGC1 activity by ~90%, consistent with a direct regulatory effect on the carrier [3]. Structural studies on the regulatory domain further support Ca^2+^-driven conformational changes that underpin this control [34]. The mitochondrial membrane potential (ΔΨ) substantially shapes AGC1 transport. Imposing K^+^ gradients in the presence of valinomycin reduced the physiological aspartate/glutamate exchange rate by ~61.5% and enhanced a non-physiological mode characterized by glutamate efflux coupled to aspartate influx, supporting a key role for ΔΨ in controlling transport directionality and efficiency [30].

Clinically, pathogenic *SLC25A12* variants impair mitochondrial aspartate transport and MAS function and are associated with global cerebral hypomyelination, leading to developmental and epileptic encephalopathy 39 (DEE39; formerly EIEE39), which is characterized by refractory seizures and severe neurodevelopmental impairment [1]. These features highlight the essential role of AGC1 in central nervous system (CNS) metabolism and myelin-related pathways [1,30].

In *Drosophila melanogaster*, current evolutionary and functional data support the presence of a single AGC gene, *aralar1/CG2139*, on chromosome 3R (cytogenetic bands 99F4–99F5), rather than two separate mammalian-like genes corresponding to *SLC25A12* and *SLC25A13* [35]. Multiple AGC isoforms arise by alternative splicing of the aralar1 gene on chromosome 3R (cytogenetic bands 99F4–99F5) [26,35]. This splicing yields six isoforms (aralar1-RA to -RF), a relatively rare phenomenon among mitochondrial carriers [36]. Constitutive isoforms (RA, RD, RF) are broadly expressed, whereas developmentally regulated isoforms (RB, RC, RE) show stage- and/or tissue-specific expression, suggesting isoform-specific regulation aligned with developmental metabolic demands [26,35]. Functional characterization has focused on Aralar1-PA and Aralar1-PE. Aralar1-PE contains a 12–amino acid insertion within the loop of its eighth EF-hand, potentially influencing transport properties. Aralar1-PA closely matches human AGC1 and catalyzes electrogenic glutamate/aspartate exchange (K_m_ 0.26 ± 0.03 mM for glutamate; 47 ± 2.3 µM for aspartate; V_max_ 32.25 ± 1.69 nmol/min/mg protein) with Ca^2+^-dependent activation (≈ 0.34 µM). Differences between Aralar1-PA and -PE appear to stem mainly from N-terminal features; the 12 extra residues in Aralar1-PE enhance transport of substrates such as cysteinesulfinate, L-α-aminoadipate, and L-glutamine, and deletion of these residues reduces activity toward Aralar1-PA levels [35]. More broadly, aralar1 activity couples intracellular Ca^2+^ signals to mitochondrial metabolism and influences glutamate availability, linking AGC function to GABA synthesis [37]. Notably, neither mammalian nor Drosophila AGC1 has been shown to directly transport GABA, and the molecular identity of the mitochondrial GABA transporter remains unresolved [38].

In *Saccharomyces cerevisiae*, Agc1p is the sole mitochondrial aspartate–glutamate carrier and functions independently of calcium due to the absence of EF-hand motifs [39]. Agc1p maintains constant activity driven by the mitochondrial membrane potential and transmembrane pH gradient [40]. It displays dual functionality, acting as both an antiporter and a uniporter, which is unique compared to its mammalian and Drosophila counterparts [39,40,41]. Kinetic analyses reveal a V_max_ of 29.4 ± 2.6 mmol/min/g protein for aspartate/aspartate exchange and 18.7 ± 1.5 mmol/min/g protein for glutamate/aspartate exchange, with K_m_ values of 0.25 ± 0.04 mM for glutamate and 40 ± 5 µM for aspartate [39]. Loss of Agc1p leads to severe metabolic disruptions, including reduced ATP production and growth defects on acetate [39,40].

Collectively, these findings highlight conserved core functions of AGC1 in redox balance and energy metabolism, while emphasizing species-specific adaptations such as calcium independence and dual transport functionality in yeast.

## 3. Clinical Recognition, Phenotypic Spectrum, and Current Management of AGC1 Deficiency

Clinically, AGC1 deficiency is characterized by early-onset pharmacoresistant seizures, severe neurodevelopmental impairment, and frequent hypomyelination, often accompanied by reduced brain N-acetylaspartate (NAA) on ^1^H-MRS [1]. The adoption of whole-exome sequencing (WES) in the diagnostic workup of pediatric mitochondrial and neurodevelopmental disorders has accelerated case identification, positioning *SLC25A12* among nuclear-encoded mitochondrial genes implicated in early-onset encephalopathies [42]. Collectively, published reports support AGC1 deficiency as a primarily neuronal disorder with variable, often secondary, white matter involvement, and these features provide a strong rationale for metabolic interventions aimed at improving bioenergetic flexibility and partially compensating for MAS-related constraints (Table 2 and Figure 2B,C).

Across published cases, the phenotype ranges from epilepsy-dominant presentations with initially unremarkable MRI [43] to extremely severe neonatal-onset epileptic encephalopathy with progressive atrophy and persistent hyperlactatemia [44].

Intermediate forms are characterized by infantile-onset seizures, hypotonia evolving into spasticity and/or movement disorders, reduced brain volume, hypomyelination, and markedly reduced NAA on ^1^H-MRS, sometimes with cerebral lactate [45,46]. A particularly relevant finding emerges from the longitudinal imaging [45], indicating that myelination may partially progress over time despite worsening cortical atrophy, consistent with secondary white matter involvement.

Additional reports [47] further suggest that AGC1 deficiency may present with predominant CNS disease even in the absence of marked systemic biochemical hallmarks such as hyperlactatemia. Detailed single-patient reports further refine the diagnostic and neuroradiological hallmarks of AGC1 deficiency. Pfeiffer [48] described a 21-month-old male (homozygous c.1331C>T; p.Thr444Ile) with initially preserved development followed by pharmacoresistant epilepsy and developmental arrest with severe hypotonia [48]. A brain MRI showed diffuse cortical atrophy with age-appropriate myelination, and ^1^H-MRS showed reduced NAA with increased myo-inositol/choline, without a lactate peak, alongside mild intermittent lactic acidemia interpreted as secondary to MAS dysfunction. A partly overlapping phenotype, but with more prominent white matter involvement, was reported by Wibom [1] in a female patient (homozygous c.1769A>G; p.Gln590Arg) with early psychomotor delay and seizures evolving to severe hypotonia and later generalized spasticity. MRI documented persistent hypomyelination with supratentorial volume loss, and ^1^H-MRS showed markedly reduced NAA, with functional assays supporting selective MAS impairment. Functional studies reinforced causality, showing elevated plasma lactate with normal amino acids, reduced ATP production with aspartate/glutamate-dependent substrates despite normal respiratory chain activities, and liposome reconstitution indicating a correctly inserted but inactive mutant AGC1, consistent with selective malate–aspartate shuttle dysfunction. In this same patient, Dahlin [49] subsequently detailed the pre-treatment course, documenting profound psychomotor arrest with absent language/interaction and markedly reduced voluntary motor activity, together with pharmacoresistant epilepsy, MRI evidence of global hypomyelination and reduced supratentorial volume, and severely reduced NAA/creatine ratio (NAA/Cr), strengthening the concept of a neuronal primary defect with secondary myelination impairment [49]. A major expansion and systematization of the phenotype comes from the cohort described by Bølsterli [50], reporting six individuals (AGC1-1–AGC1-6) who share seizure onset within the first year of life, often focal, apnea-associated, and pharmacoresistant severe neurodevelopmental impairment with arrest/regression, early hypotonia frequently evolving into spasticity and/or movement disorders, and frequent feeding difficulties (often requiring enteral feeding), with secondary microcephaly/failure to thrive in some cases [50]. Neuroradiological hallmarks include recurrent supratentorial volume loss and diffuse hypomyelination (sometimes interpreted as secondary), while metabolic findings include intermittent lactate elevations; where available, ^1^H-MRS shows reduced NAA with increased myo-inositol, with intermittent cerebral lactate peaks in AGC1-1 and notably low cerebrospinal fluid aspartate in AGC1-1 despite normal plasma values.

**Figure 3:**
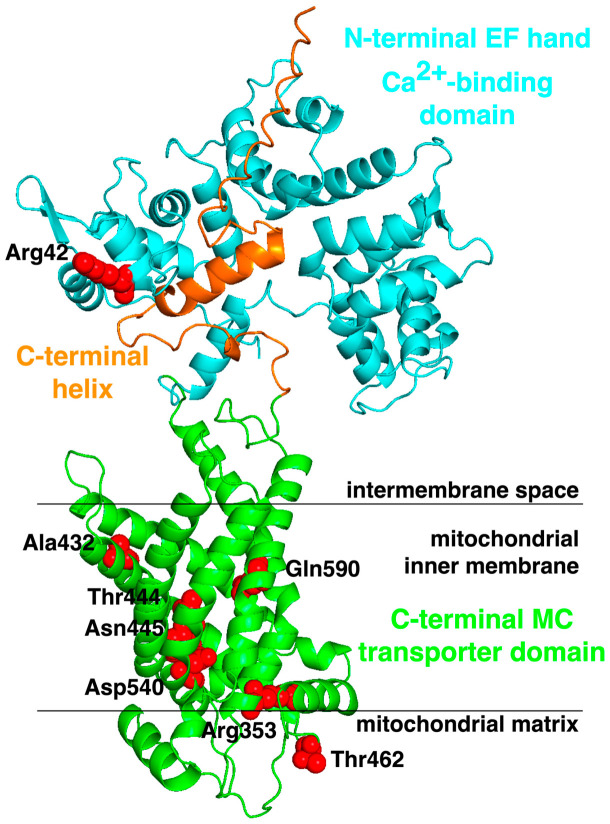
The pathogenic missense mutations mapped in the structural model of human AGC1. The pathogenic mutants are indicated with red spheres in the model of AGC1 (AF-O75746-F1) from the AlphaFold DB [51]. The N-terminal EF-hand Ca^2+^-binding regulatory domain is shown in cyan, the C-terminal helix in orange, and the mitochondrial carrier domain in green. The approximate positions of the intermembrane space, mitochondrial inner membrane, and mitochondrial matrix are indicated.

From a management perspective, treatment remains largely symptomatic, relying on antiepileptic drugs and supportive care, and seizure control often dissociates from neurodevelopmental recovery. Given the absence of established disease-modifying therapies, metabolic interventions have been explored as rational adjuncts. Among these, the ketogenic diet (KD) has been most systematically reported and is discussed in detail in the following section. To explore whether the molecular consequences of reported *SLC25A12* variants may help interpret disease severity, we mapped the pathogenic missense variants onto the predicted AGC1 structural model (Figure 3 and Table 2).

Because the number of reported patients remains small and the clinical spectrum is heterogeneous, no definitive genotype-phenotype correlation can currently be established. However, a cautious functional stratification can be proposed. Notably, most currently reported pathogenic variants affect coding exons, whereas the main non-coding exceptions are splice-disrupting changes. Variants predicted to abolish or severely impair AGC1 function include the nonsense variant p.Arg134*, the frameshift variants p.Leu271Thrfs*9 and p.Glu76Serfs*17, the deletion of exons 16 and 17, and the synonymous variant p.Arg583Arg, which is predicted to affect splicing. Likewise, the combined p.Ala432Val + c.1447-2_1447-1delAG genotype is overall predicted to be deleterious, although the individual contribution of each change cannot be clearly separated. In contrast, missense variants may have variable effects depending on their structural location. Variants exposed toward or near the substrate translocation pathway, including p.Thr444Ile, p.Asp540Asn, and p.Gln590Arg, may more directly interfere with transport activity, whereas buried variants, such as p.Arg353Gln and p.Asn445Lys, may affect protein folding, structural stability, or intramolecular interactions. By contrast, the functional consequences of p.Arg42Pro, located in the intermembrane-space-facing region, and p.Thr462Met, exposed toward the matrix, are more difficult to predict from the structural model alone. Therefore, structural mapping supports the pathogenic interpretation of these variants but does not yet allow robust prediction of mild versus severe clinical outcome. The structural localization and predicted functional interpretation of these variants are summarized in Table 2.

**Table 2:** Genetic variants, structural protein localization, clinical features, diagnostic findings, treatment, and outcomes in reported patients with AGC1 deficiency.

Genetic Mutations and Zygosity	Protein Impact and Position in the Structural Model *	Symptomatology	Diagnosis	Treatment	Clinical, Diagnostic, and Treatment Outcome	Ref.
NM_003705.5:c.1385C>T Homozygous	p.Thr462Met missense Exposed in the matrix	Early-onset epileptic encephalopathy, GDD, hypotonia/spasticity, poor visual tracking	WES	AEDs	Refractory seizures despite AEDs; severe neurodevelopmental impairment.	[43]
NM_003705.5:c.125G>C Homozygous	p.Arg42Pro missense Exposed in the intermembrane space	Early-onset neurological disease, GDD, hypotonia	WES	n.d.a.	n.d.a.	[47]
NM_003705.5:c.400C>T Homozygous	p.Arg134Ter nonsense not applicable	Severe GDD, absent speech, hypertonia/CP, infantile-onset refractory seizures, hyperlactatemia	WES; MRI; hyperlactatemia with otherwise unremarkable metabolic workup.	Pre-existing AEDs therapy	Persistent seizures and severe deficits despite AEDs; fatal outcome.	[44]
NM_003705.5:c.1295C>T; NC_000002.12(NM_003705.5):c.1447-2_1447-1delAG Compound heterozygous	p.Ala432Val missense; buried between transmembrane helices intronic deletion affecting splicing	Severe DD, hypotonia, epilepsy, spastic quadriplegia	WES, MRI, MRS	AEDs polytherapy; later levetiracetam reintroduced	Seizure control with AED polytherapy; persistent neurodevelopmental impairment.	[45]
NM_003705.5:c.1058G>A Homozygous	p.Arg353Gln missense buried towards the matrix	GDD, congenital hypotonia, infantile-onset epilepsy, absent speech, severe motor impairment	WES; MRI/MRS showing cerebral atrophy, reduced NAA, with a focal lactate peak; muscle biopsy	Topiramate, Phenobarbital	Seizure control with AEDs; persistent severe impairment; abnormal MRS.	[46]
NM_003705.5:c.1058G>A Homozygous	p.Arg353Gln missense buried towards the matrix	Infantile-onset epilepsy, severe GDD	WES	n.d.a.	Limited clinical data available.	[46]
NM_003705.5:c.1331C>T Homozygous	p.Thr444Ile missense exposed in the substrate translocation pore	Refractory epilepsy, developmental arrest, generalized hypotonia, limited speech	WES, MRI: age-appropriate myelination, MRS: no lactate peak, Biochemical workup	Multiple AEDs + KD (4:1)	Seizure freedom within 4 months of KD; improved head/neck control; persistent DD and reduced NAA.	[48]
NM_003705.5:c.1769A>G Homozygous	p.Gln590Arg missense exposed in the substrate translocation pore	Severe hypotonia, seizures, psychomotor delay	WES, MRI, MRS, Biochemical workup	AEDs, supportive care, KD at six years	After KD, seizure freedom, resumed myelination, increased brain volume/NAA, and AED tapering.	[1,49]
NM_003705.5:c.1586-?_1835+?del (AGC1-1) Homozygous	Deletion of exons 16 and 17, exact break-point not determined	Refractory epilepsy, severe DD, hypotonia	WES, MRI, MRS	Multiple AEDs; cKD at 1y9m	Marked KD response: seizure freedom and improved MRI myelination.	[50]
NM_003705.5:c.1618G>A (AGC1-2) Homozygous	p.Asp540Asn missense exposed in the substrate translocation pore	Early-onset epilepsy, severe GDD, hypotonia/spasticity, regression	WES, MRI, MRS	AEDs + KD (2:1) at ~5y8m	Modest seizure improvement on KD; no motor/developmental gains.	[50]
NM_003705.5:c.1618G>A (AGC1-3) Homozygous	p.Asp540Asn missense exposed in the substrate translocation pore	DD, seizures, hypotonia, regression	WES, MRI, MRS	KD, AEDs	Seizure improvement on KD; persistent hypotonia and neurodevelopmental impairment.	[50]
NM_003705.5:c.810_811insA (AGC1-4) Homozygous	p.Leu271ThrfsTer9 frameshift	Severe DD, dystonia-spasticity, epilepsy	WES, MRI, MRS	KD, supportive care	No meaningful KD benefit; persistent severe DD, dystonia-spasticity, and epilepsy.	[50]
NM_003705.5:c.225del; NM_003705.5:c.1747C>A (AGC1-5) Compound heterozygous	p.Glu76SerfsTer17 frameshift; p. (=) synonymous variant affecting splicing	Refractory epilepsy, severe hypotonia, regression	WES, MRI, MRS	KD, AEDs	Marked KD response: seizure reduction and later independent walking; stable deficits.	[50]
NM_003705.5:c.1335C>A (AGC1-6) Homozygous	p.Asn445Lys missense buried between transmembrane helices	Severe neurological impairment, epilepsy, GDD	WES, MRI	KD, AEDs, supportive therapies	Marked seizure improvement with AEDs/KD; persistent severe neurodevelopmental impairment.	[50]

Included in this table are all published reports of patients with AGC1 deficiencies. The table also includes genotype, predicted protein impact, clinical phenotype, diagnostic findings, treatment, and outcomes. Each row corresponds to a single patient analyzed per study; in the study by Bolsterli et al. [50], six previously unreported AGC1-deficient cases were described and are presented individually. In the study by Falk et al. [46], the two analyzed patients were siblings; for one of them, the affected sibling was reported to have a clinical course similar to the proband, with no imaging or treatment details provided. All *SLC25A12* variants are reported according to HGVS nomenclature using the reference transcript NM_003705.5. Protein-level consequences are reported using the three-letter amino acid code. Variants were checked, where possible, using Mutalyzer 3 “https://mutalyzer.nl/ (accessed on 08 May 2026)”. For exon-level deletions with undetermined breakpoints, such as NM_003705.5.1586-?_1835+?del, full genomic normalization was not possible because the exact breakpoints were not available in the original report; these variants are therefore retained as published and annotated as breakpoint-undetermined deletions. * For the structural model, see Figure 3. Abbreviations: AGC1, aspartate–glutamate carrier 1; AEDs, antiepileptic drugs; CP, cerebral palsy; cKD, classical ketogenic diet; DD, developmental delay; GDD, global developmental delay; KD, ketogenic diet; MRI, magnetic resonance imaging; MRS, magnetic resonance spectroscopy; NAA, N-acetylaspartate; WES, whole-exome sequencing; n.d.a., not data available.

## 4. The Ketogenic Diet as a Therapeutic Approach in AGC1 Deficiency: Mechanisms, Clinical Evidence, and Perspectives

The ketogenic diet (KD) is an established non-pharmacological therapy for drug-resistant epilepsy and has also been explored as a targeted metabolic strategy in AGC1 deficiency. KD is a high-fat, carbohydrate-restricted regimen that induces ketosis, thereby increasing circulating ketone bodies—primarily β-hydroxybutyrate (BHB) and acetoacetate. These ketones cross the blood–brain barrier and serve as alternative oxidative substrates for the brain. In pediatric epilepsy, KD efficacy is supported by controlled clinical trials reporting significant seizure reductions compared with conventional care [52,53,54]. Beyond seizure control, KD has been associated with broader neuroprotective effects, including modulation of neurotransmission, reduced oxidative stress, and attenuation of inflammatory pathways [55,56], as well as improvements in mitochondrial function and cerebral bioenergetics across several neurological contexts [57,58,59].

Multiple mechanisms have been proposed to contribute to KD efficacy, although their relevance to AGC1 deficiency is likely heterogeneous and should be interpreted as plausible contributors rather than strictly AGC1-specific pathways. First, ketone bodies may support mitochondrial bioenergetics and enhance metabolic robustness in settings where MAS-dependent redox balancing is impaired, potentially influencing downstream metabolites linked to neuronal integrity (including NAA) and myelination-associated processes. Second, KD may reduce neuronal hyperexcitability by shifting the excitatory/inhibitory balance, in part through effects on glutamate–GABA metabolism and inhibitory neurotransmission [53,60,61]. Third, ketone bodies can modulate transcriptional and inflammatory signaling, including histone deacetylase (HDAC) inhibition, reduced pro-inflammatory mediators, and increased neurotrophic factors (e.g., BDNF, NGF), which may support synaptic plasticity and, potentially, myelination [62]. In the same human model, AGC1-deficient neuronal progenitors display extensive transcriptomic remodeling and altered histone acetylation/methylation profiles, supporting a metabolic–epigenetic coupling [63]. Finally, KD-associated changes in gut microbiota composition have been proposed to affect the gut–brain axis and systemic/neuroinflammatory tone [58]. More broadly, defects in SLC-family metabolite transporters, including *SLC25A12*, are increasingly recognized among genetic epileptic encephalopathies and neurodevelopmental disorders, underscoring the contribution of impaired metabolite transport to seizure phenotypes [64,65] (Figure 2D).

AGC1-specific clinical evidence is limited but clinically informative. Published KD-treated cases include those reported by Pfeiffer, Dahlin, and the cohort described by Bølsterli, with overall promising yet variable outcomes [48,49,50] (Table 2). In the patient described by Pfeiffer [48], KD initiation for pharmacoresistant epilepsy was associated with a clinically meaningful reduction in seizure frequency and improved alertness and interaction. However, the absence of neuroradiological or spectroscopic follow-up and the lack of documented recovery of major developmental milestones suggest predominantly symptomatic benefit. In contrast, Dahlin et al. reported a more robust and progressive response, including seizure remission, EEG normalization, recovery of psychomotor skills, and objective evidence of structural and metabolic improvement, namely, resumption of myelination, increased brain volume, and increased NAA on ^1^H-MRS, supporting the possibility that KD may influence CNS maturation in at least a subset of patients.

Across reported cases, seizure reduction emerges as the most reproducible benefit, whereas changes in neurodevelopment, movement disorders, and MRI/^1^H-MRS parameters appear more heterogeneous. In the Bølsterli cohort [50], marked improvements, including increased NAA and progression of myelination, were reported in some individuals (e.g., AGC1-1 and AGC1-5), whereas later initiation or unstable ketosis was associated with more limited functional gains. Interpretation across studies remains constrained by variability in follow-up depth, including missing MRI/^1^H-MRS in some reports, and by differences in ketosis stability.

Parallel experimental studies strengthen the translational rationale and suggest that BHB itself may recapitulate part of KD benefits. In cell and mouse models, direct BHB treatment preserved mitochondrial respiration, supported synthesis of key metabolites (including aspartate and NAA), promoted myelination, and protected AGC1-deficient neurons from glutamatergic excitotoxicity [20,66]. Notably, these studies also reported practical limitations in applying KD to AGC1 knockout mice, motivating the exploration of ketone-based strategies as alternatives or adjuncts. Additional approaches, such as epigenetic modulation with curcumin, have shown mixed in vitro effects on neuronal and oligodendrocyte precursor populations and therefore require cautious interpretation and further preclinical validation [67].

Safety monitoring is essential. Systematic reviews and meta-analyses document adverse events, including dyslipidemia, kidney stones, gastrointestinal symptoms, and nutritional deficiencies [68,69]. Less restrictive dietary variants, such as medium-chain triglyceride-based regimens or the Modified Atkins Diet, may improve tolerability and adherence [70]. Overall, responses to KD in AGC1 deficiency are heterogeneous and likely influenced by age at initiation, duration and stability of ketosis, baseline disease severity, and genotype. While current evidence supports KD as a relevant therapeutic option—particularly when initiated early—available data remain insufficient to determine whether KD is consistently disease-modifying or primarily provides a variable combination of metabolic support and seizure suppression. Prospective longitudinal studies with standardized clinical endpoints integrated with MRI/^1^H-MRS and metabolic biomarkers are needed to define predictors of response and optimize individualized protocols.

## 5. *SLC25A12* and Autism Spectrum Disorder: A Candidate Gene with Conflicting Evidence

Several genetic association studies have investigated *SLC25A12*, encoding the mitochondrial aspartate–glutamate carrier AGC1, as a candidate susceptibility gene for autism spectrum disorder (ASD). Early linkage and association reports suggested an association between common variants (e.g., SNPs rs2056202 and rs2292813) and ASD-related traits, including restricted, repetitive behaviors and social impairment [71,72]. Complementary post-mortem and experimental studies proposed that altered calcium signaling, mitochondrial dysfunction, and impaired axonal transport involving AGC1 could contribute to ASD-relevant neurobiology [73]. However, subsequent studies have yielded mixed results, and larger cohorts as well as meta-analyses have not consistently supported a significant association between *SLC25A12* common variants and ASD; moreover, AGC1 expression measures in brain tissue have not shown a robust relationship with ASD severity [29,74]. Overall, while *SLC25A12* may have been considered a candidate gene for ASD, the available evidence remains inconclusive and substantially less robust than its established role in severe neurodevelopmental disease caused by biallelic pathogenic variants in *SLC25A12*.

## 6. Conclusions and Perspectives

The aspartate–glutamate carrier AGC1 (*SLC25A12*) is a significant mitochondrial transporter that links cytosolic redox balance and nitrogen flux to neuronal bioenergetic demands through the malate–aspartate shuttle (MAS). Its calcium-dependent regulation and tissue-enriched expression help explain why AGC1 dysfunction preferentially impacts the developing brain, where it contributes to synaptic activity, neuronal integrity, and myelination-related metabolism. Comparative analyses across mammals, *Drosophila melanogaster*, and *Saccharomyces cerevisiae* underscore conserved transport principles while revealing organism-specific adaptations that refine our understanding of how mitochondrial carriers are integrated into broader metabolic networks.

Clinically, biallelic pathogenic variants in *SLC25A12* cause a severe developmental and epileptic encephalopathy, commonly referred to as DEE39, characterized by early-onset pharmacoresistant seizures, profound neurodevelopmental impairment, and recurrent neuroimaging signatures that frequently include hypomyelination and markedly reduced N-acetylaspartate (NAA) on ^1^H-MRS. Across published reports, seizure control often dissociates from neurodevelopmental outcome under standard symptomatic management, supporting the need for mechanism-informed adjunctive strategies. In this context, the ketogenic diet (KD) represents the most consistently reported metabolic intervention. Available clinical evidence suggests that seizure reduction is the most reproducible benefit, whereas neurodevelopmental and MRI/^1^H-MRS improvements, including increases in NAA and progression of myelination, are more heterogeneous and appear influenced by timing, duration, and stability of ketosis. Preclinical studies further strengthen the translational rationale by showing that β-hydroxybutyrate can preserve mitochondrial respiration, support metabolite synthesis, and promote myelination in AGC1-deficient models, motivating exploration of ketone-based approaches alongside dietary therapy. Prospective longitudinal studies integrating standardized clinical outcomes with mechanistically linked MRI/^1^H-MRS measures and metabolic readouts will be essential to define predictors of response and clarify the extent to which KD and related strategies can be disease-modifying.

In contrast, the proposed association between *SLC25A12* common variants and autism spectrum disorder remains inconclusive. While early genetic studies suggested links with ASD-related traits, subsequent analyses have produced inconsistent results, and current evidence does not support *SLC25A12* as a primary ASD risk gene. Overall, AGC1 exemplifies how integrating molecular mechanisms, comparative biology, and human genetics can refine disease classification and guide therapeutic development, while highlighting mitochondrial metabolite transport as a tractable axis for intervention in neurodevelopmental encephalopathies.

## Figures and Tables

**Table 1:** Key differences between the two mammalian aspartate-glutamate carriers.

Features	AGC1/Aralar	AGC2/Citrin	References
Transport properties	Same substrate specificity; similar K_m_	Same substrate specificity; similar K_m_	[3]
Ca^2+^ sensitivity	Lower	Higher	[13]
Main tissue distribution	Brain, skeletal muscle, heart	Liver, non-excitable tissues, heart	[14,24,26,27]
Main physiological emphasis	Neuronal MAS activity, NAA synthesis, myelination	Urea-cycle support, gluconeogenesis, hepatic redox balance	[16,21,22,24]
Disease association	Predominantly neurological phenotype with epilepsy and hypomyelination	Predominantly hepatic/metabolic phenotype with citrin deficiency	[1,21,22,23]

## Data Availability

No new data were created or analyzed in this study. Data sharing is not applicable to this article.

## Abbreviations

The following abbreviations are used in this manuscript:

^1^H-MRS	Proton magnetic resonance spectroscopy
AGC1/2	Aspartate/glutamate carrier 1/2
ASD	autism spectrum disorder
BHB	β-hydroxybutyrate
CNS	central nervous system
DEE39	developmental and epileptic encephalopathy 39
EEG	electroencephalogram
GOT1/2	glutamic-oxaloacetic transaminases
HDAC	histone deacetylase
KD	ketogenic diet
MAS	Malate-aspartate shuttle
MC	mitochondrial carrier
MDH1/2	malate dehydrogenases
MRI	Magnetic Resonance Imaging
NAA	N-acetylaspartate
NAA/Cr	NAA/creatine ratio
SLC25	solute carrier family 25
WES	whole exome sequencing
